# Rapid cleavage of RNA by RNase E in the absence of 5′ monophosphate stimulation

**DOI:** 10.1111/j.1365-2958.2009.06935.x

**Published:** 2009-11-18

**Authors:** Louise Kime, Stefanie S Jourdan, Jonathan A Stead, Ana Hidalgo-Sastre, Kenneth J McDowall

**Affiliations:** Astbury Centre for Structural Molecular Biology, Faculty of Biological Sciences, University of LeedsLS2 9JT, England, UK

## Abstract

The best characterized pathway for the initiation of mRNA degradation in *Escherichia coli* involves the removal of the 5′-terminal pyrophosphate to generate a monophosphate group that stimulates endonucleolytic cleavage by RNase E. We show here however, using well-characterized oligonucleotide substrates and mRNA transcripts, that RNase E can cleave certain RNAs rapidly without requiring a 5′-monophosphorylated end. Moreover, the minimum substrate requirement for this mode of cleavage, which can be categorized as ‘direct’ or ‘internal’ entry, appears to be multiple single-stranded segments in a conformational context that allows their simultaneous interaction with RNase E. While previous work has alluded to the existence of a 5′ end-independent mechanism of mRNA degradation, the relative simplicity of the requirements identified here for direct entry suggests that it could represent a major means by which mRNA degradation is initiated in *E. coli* and other organisms that contain homologues of RNase E. Our results have implications for the interplay of translation and mRNA degradation and models of gene regulation by small non-coding RNAs.

## Introduction

*Escherichia coli* RNase E is required for the normal rapid degradation of many, if not most transcripts (for recent reviews, see Deutscher, 2006; Carpousis *et al.*, 2009), including RNAI, the antisense RNA regulator of ColEl-type plasmid replication (Tomcsanyi and Apirion, 1985; Lin-Chao and Cohen, 1991). The major site of RNase E cleavage in RNAI is located in a single-stranded region at the 5′ end (Tomcsanyi and Apirion, 1985; Lin-Chao and Cohen, 1991; McDowall *et al.*, 1994). Oligonucleotide substrates based on this site (e.g. BR13; McDowall *et al.*, 1995) have been used to study the function and structure of members of the RNase E family (for recent reviews, see Kime *et al.*, 2008; Carpousis *et al.*, 2009). *E. coli* RNase E also has a role in the processing of precursors of ribosomal RNA (Ghora and Apirion, 1978; Misra and Apirion, 1979; Li *et al.*, 1999) and transfer RNA (Li and Deutscher, 2002; Ow and Kushner, 2002) as well as several small, non-coding RNAs (Lundberg and Altman, 1995; Lin-Chao *et al.*, 1999). Concordant with its central role in RNA processing and degradation, RNase E is essential for *E. coli* viability (Apirion and Lassar, 1978; Ono and Kuwano, 1979). RNase E is assisted in the generation of 16S rRNA by its paralogue, RNase G (Li *et al.*, 1999; Wachi *et al.*, 1999). The latter is also required for the normal degradation of many transcripts (Lee *et al.*, 2002), including functional forms of *adhE* and *eno* mRNA (Wachi *et al.*, 2001; Kaga *et al.*, 2002; Jourdan and McDowall, 2008).

The domains of *E. coli* RNase E that are required for catalysis are found in its N-terminal half (NTH) (McDowall and Cohen, 1996; Callaghan *et al.*, 2005a; Caruthers *et al.*, 2006), which is similar in sequence to RNase G (McDowall *et al.*, 1993). The NTH of RNase E forms a tetramer (Callaghan *et al.*, 2003), which is a dimer of the dimeric unit that forms the active sites (Callaghan *et al.*, 2005a). Segments that appear to stabilize the dimer : dimer interface in *E. coli* RNase E are lacking in RNase G explaining why the latter is found predominantly as a dimer (Briant *et al.*, 2003; Jourdan and McDowall, 2008). The C-terminal half of RNase E contains ancillary RNA-binding sites (Taraseviciene *et al.*, 1995; McDowall and Cohen, 1996; Kaberdin *et al.*, 2000) and segments that facilitate interaction with the inner membrane (Miczak *et al.*, 1991; Liou *et al.*, 2001; Khemici *et al.*, 2008) and with other enzymes as part of a complex called the RNA degradosome (for reviews, see Carpousis *et al.*, 2001; 2009; Carpousis, 2002; 2007; Marcaida *et al.*, 2006). However, only domains within the NTH of *E. coli* RNase E are required for cell viability (Kido *et al.*, 1996; Jiang *et al.*, 2000).

The study of RNAI has revealed that it can be stabilized *in vivo* by adding a stem-loop such that the extreme 5′ end presents a single-stranded segment of no more than two to four nucleotides (Bouvet and Belasco, 1992). Such structures had been shown previously to be responsible for the stability of the *E. coli ompA* transcript (Emory *et al.*, 1992), which with a half-life of 15–20 min is one of the most stable *E. coli* mRNAs (von Gabain *et al.*, 1983). This indicated that, despite being an endonuclease, RNase E is sensitive to the structure at the 5′ end of at least some transcripts *in vivo* (Bouvet and Belasco, 1992). In another study, it was found that the rate of RNase E cleavage of *rpsT* mRNA and the 9S precursor of 5S rRNA *in vitro* was most rapid when the 5′ end was both single-stranded and terminated with a monophosphate group; forms that were circular or had a triphosphate group or base pairing at the extreme 5′ end were less susceptible to RNase E (Mackie, 1998). Later it was shown for *rpsT* mRNA that the addition of a 5′ stem-loop or circularization also result in stabilization in *E. coli* (Mackie, 2000; Baker and Mackie, 2003).

More recently, the solving of the X-ray crystal structures of the catalytic domain of *E. coli* RNase E in complexes with single-stranded oligonucleotide substrates revealed a pocket that can bind a 5′ monophosphate and residues in an RNA-binding channel that contact the first nucleotides; furthermore, modelling indicated that these structures cannot accommodate 5′ ends that have either a triphosphate group or nucleotides that are base-paired (Callaghan *et al.*, 2005a). While this study offered an explanation for the preference of RNase E for 5′-monophosphorylated versions of both oligonucleotide substrates (Tock *et al.*, 2000) and transcripts such as 9S RNA and *rpsT* mRNA *in vitro* (Mackie, 1998), it remained unclear as to how 5′ stem-loops provided transcripts such as RNAI and *rpsT* mRNA with increased protection against RNase E-mediated degradation *in vivo* (Bouvet and Belasco, 1992; Baker and Mackie, 2003) given that primary transcripts are synthesized with a 5′ triphosphate group. This was resolved recently with the discovery in *E. coli* of a 5′ pyrophosphatase (now called RppH) that converts the 5′ group of primary transcripts from a triphosphate to a monophosphate (Celesnik *et al.*, 2007; Deana *et al.*, 2008).

The study of selected transcripts in *E. coli* revealed that in the absence of RppH the half-life of *rpsT* mRNA increases about fourfold confirming the importance under normal physiological conditions of generating a monophosphate on its single-stranded 5′ end (Deana *et al.*, 2008). However, on a genome-wide scale, the cellular levels of the majority of transcripts in *E. coli* seemed to be largely unaffected by deleting RppH (Deana *et al.*, 2008). Thus, much of the mRNA degradation in *E. coli* appears to proceed without primary transcripts needing to have a 5′-monophosphorylated end. Prior to the characterization of RppH, there had been speculation that RNase E might be able to initiate mRNA degradation without interacting with a 5′ end. This was called the ‘direct entry’ model (for review, see Coburn and Mackie, 1999). However, the evidence was limited to an example that had not been shown to be highly dependent on RNase E for its degradation (Hansen *et al.*, 1994) and one where the transcript had been engineered to be highly stable (Baker and Mackie, 2003).

Recently we reported for RNase G that, in contrast to a study by others (Jiang and Belasco, 2004), a 5′ monophosphate is not crucial for ‘catalytic activation’ and instead enhances the affinity of RNase G binding to RNA (Jourdan and McDowall, 2008). Here, using derivatives of BR13 (McDowall *et al.*, 1995), we provide evidence that multiple single-stranded regions in a conformational context that allows their simultaneous interaction with RNase E can produce a complex that is sufficiently stable to negate the requirement for a 5′ monophosphate group. Moreover, we demonstrate that *cspA* mRNA, a known RNase E substrate (Hankins *et al.*, 2007) that appears not to be affected by disruption of RppH (Deana *et al.*, 2008) is cleaved rapidly irrespective of the phosphorylation status at its 5′ end. This substrate is shown to contain multiple single-stranded sites that can be recognized by RNase E. Given the frequency of occurrence of single-stranded segments in mRNA, we propose that the direct entry of RNase E to internal sites may represent a major pathway by which degradation is initiated in *E. coli*.

## Results

### Rapid cleavage of a 5′-hydroxylated substrate

**Fig. 1:**
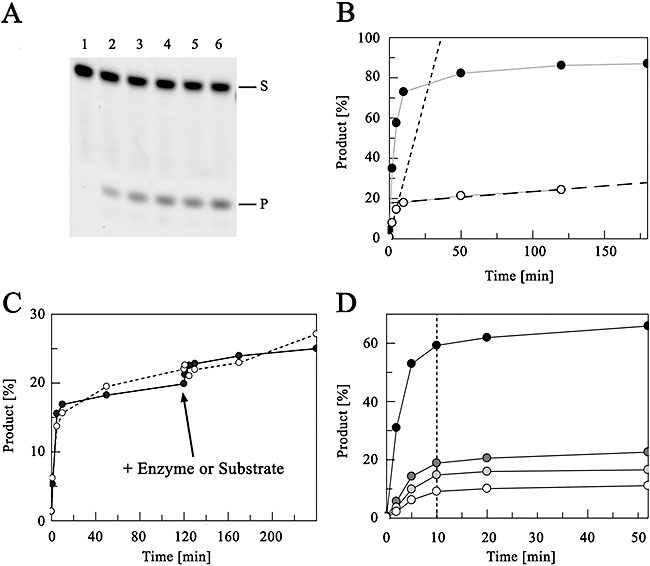
Cleavage of 5′-hydroxylated oligonucleotide substrate by the N-terminal half of RNase E. A. The cleavage of 5′-hydroxylated BR13 (McDowall *et al.*, 1995; Tock *et al.*, 2000) labelled at the 3′ end with fluorescein. The reaction products were separated on a 15% polyacrylamide, sequencing-type gel. Lanes 1–6 contain samples taken 0, 2, 5, 10, 50 and 120 min after mixing substrate and enzyme. The reaction components were stored on ice prior to mixing. The position of bands corresponding to substrate (S) and downstream product (P) are indicated on the right of the panel. The enzyme and substrate concentrations at the start of a reaction were 5 and 250 nM respectively. B. Plots of the amount of product formed with time for BR13 that has a hydroxyl (open circles) or monophosphate (closed circles) group at its 5′ end. The conditions for cleavage of the latter were as (A). The lines with short and long dashes represent linear fits of data for the 5′-hydroxylated substrate between 0 and 5 min and 10 and 120 min respectively. C. Data for assays to which either fresh substrate (solid line) or enzyme (dotted line) was added after the reactions had reached the second, slower phase. The concentrations of enzyme and substrate at the start of the reaction were as described for (A). The addition of a second aliquot of enzyme or substrate (one-quarter volume) altered the total concentrations added to 8 nM enzyme and 200 nM substrate or 4 nM enzyme and 400 nM substrate respectively. D. Data for the cleavage of 5′-hydroxylated BR13 at an initial concentration of 250 nM by NTH-RNase E at concentrations of 1.25 nM (white circles), 2.5 nM (light grey), 5 nM (dark grey) or 25 nM (black). The vertical line with short dashes indicates the time point after which cleavage is judged to be slow.

While assaying the cleavage of 5′-hydroxylated BR13 (Tock *et al.*, 2000) by a preparation of NTH-RNase E (Fig. 1), we noticed that the reaction slowed after only a small proportion of the substrate had been cleaved (Fig. 1A). Plotting of the data suggested that there was an initial phase of ∼10 min during which the rate of cleavage was ∼40-fold higher than in the remainder of the reaction (Fig. 1B). The calculated turnover numbers for the two phases were 1.3 and 0.03 min^−1^ respectively. The rate during the first phase was similar to the initial rate of cleavage of 5′-monophosphorylated BR13 (5.2 min^−1^) under the same reaction conditions (Fig. 1B). To establish the source of the biphasic cleavage of 5′-hydroxylated BR13, aliquots of fresh enzyme or substrate were added to reactions that had reached the second, slower phase of cleavage (Fig. 1C). Addition of NTH-RNase E increased the rate, but no more than expected from increasing the enzyme concentration. In contrast, the addition of fresh substrate led to another phase of rapid cleavage. This revealed that the source of the phase of rapid cleavage was not the enzyme (e.g. burst kinetics), but the substrate. It was also found that the amount of substrate that could be cleaved during the first phase was not fixed, but was instead related to the enzyme concentration (Fig. 1D). At the highest enzyme concentration, the majority of the substrate appeared to be highly susceptible to RNase E. In all the reactions, the rates appeared to slow considerably after ∼10 min. These results suggested that initially the substrate was highly susceptible to RNase E cleavage, but over a period of minutes underwent a conversion to a less susceptible form. The above was reproducible using substrate from a separate synthesis that had also been verified using mass spectrometry (data not shown).

### Temperature-induced change in substrate conformation

**Fig. 2:**
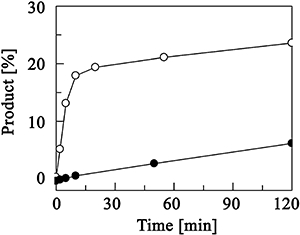
Preincubation at the reaction temperature abolishes the rapid phase of cleavage of BR13. The figure shows data for two cleavage assays using 5′-hydroxylated BR13 and RNase E at concentrations of 250 and 5 nM respectively. Open and closed circles represent data for substrate that had been stored on ice or prewarmed to the reaction temperature respectively, before combining with enzyme.

The above reactions were assembled from mixtures of enzyme and substrate that had been stored on ice prior to being combined and placed in a metal block prewarmed to the reaction temperature. To investigate whether an increase in temperature reduced the susceptibility of 5′-hydroxylated BR13 to RNase E, both the mixtures of enzyme and substrate were prewarmed to the reaction temperature for 15 min before combining. This was found to abolish the phase of rapid cleavage, as did prewarming only the substrate (Fig. 2). BR13 has a 5′-guanosine triplet followed by the decanucleotide sequence (5′-ACAGU↓AUUUG, arrow indicates major cleavage site) that corresponds to the RNase E-cleaved segment at the 5′ end of RNAI from plasmid pBR322 (Tomcsanyi and Apirion, 1985; Lin-Chao and Cohen, 1991). The 5′-guanosine triplet was incorporated for earlier studies that required the sequence to be identical to the 5′ segment of GGG-RNAI, a full-length transcript generated *in vitro* using T7 RNA polymerase (Lin-Chao *et al.*, 1994; McDowall *et al.*, 1995; McDowall and Cohen, 1996). Analysis of the BR13 sequence using PairFold (Andronescu *et al.*, 2005) and MFold (Zuker, 2003) indicated that complementary base pairing was unlikely to produce a structure that would take minutes to dissociate at 37°C (data not shown).

### Intermolecular quadruplex formation

**Fig. 3:**
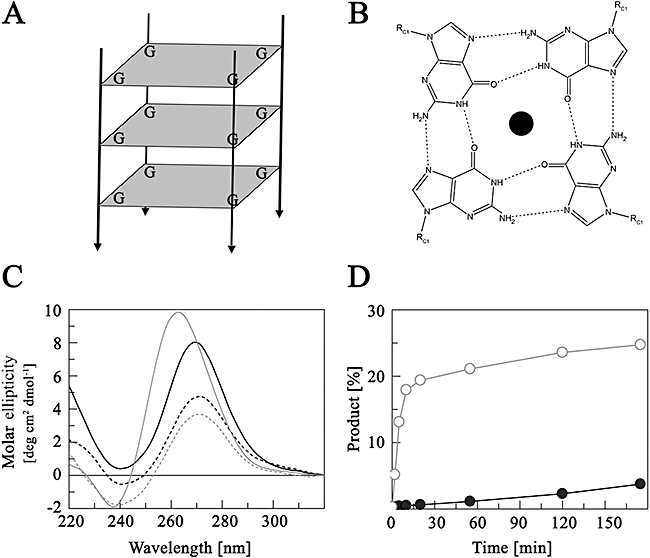
Analysis of 5′-hydroxylated BR13 using CD and enzymatic assays. A. A schematic drawing of a parallel quadruplex. B. The arrangement of four guanosines in a G-quartet. Dashed lines represent the eight hydrogen bonds formed between the four guanosines. A cation (e.g. potassium) is usually located in the middle of two quartets and is shown here as a black sphere. Taken from Burge *et al.* (2006). C. The CD spectra of BR13 (grey lines) and LU13 (black lines) at a concentration of 7.5 μM in 25 mM *bis*-Tris-Propane (pH 8.3) and 100 mM NaCl. The solid and dotted line represent data collected at 4°C and 37°C respectively. LU13 is a BR13 derivative that has the central G of the 5′ triplet replaced with an A. D. Data for the cleavage of 5′-hydroxylated BR13 (open circles) and LU13 (closed circles) as per the conditions described in Fig. 1.

Next we used circular dichroism (Fig. 3) to determine whether at low temperature the guanosine triplet at the 5′ end of BR13 was sufficient to mediate the formation of an intermolecular quadruplex (Neidle and Balasubramanian, 2006). Such structures (Fig. 3A) result from the hydrophobic stacking of G quartets (Fig. 3B), each of which is a planar arrangement of guanosines held together by hydrogen bonding that utilizes the Hoogsteen as well as the Watson-Crick face of the bases; a dehydrated sodium or potassium ion binds to the interior of the quadruplex through co-ordination of guanine O6 groups of successive tetrad stacks (Parkinson, 2006). Oligonucleotide BR13 in the presence of sodium ion at 4°C produced a spectrum that was characteristic of intermolecular quadruplexes in which the strands are parallel (Balagurumoorthy *et al.*, 1992; Lu *et al.*, 1992; 1993; Balagurumoorthy and Brahmachari, 1994); maximum negative and positive values of molecular ellipticity were obtained at ∼240 and ∼260 nm respectively (Fig. 3C). Analytical ultracentrifugation analysis was consistent with BR13 forming a quadruplex at 4°C (data not shown). Heating BR13 to 37°C resulted in a loss of the characteristic spectrum; there was a clear shift in the wavelength of the maximum positive value of molecular ellipticity to ∼270 nm (Fig. 3C). A spectrum similar to the latter was produced when quadruplex formation was disrupted by replacing the central G of the triplet at the 5′ end with an A (see substrate LU13, Fig. 3C). Moreover, this substitution abolished the phase of rapid cleavage of 5′-hydroxylated substrate by NTH-RNase E (Fig. 3D).

### Substrate multimers stimulate rapid cleavage

**Fig. 4:**
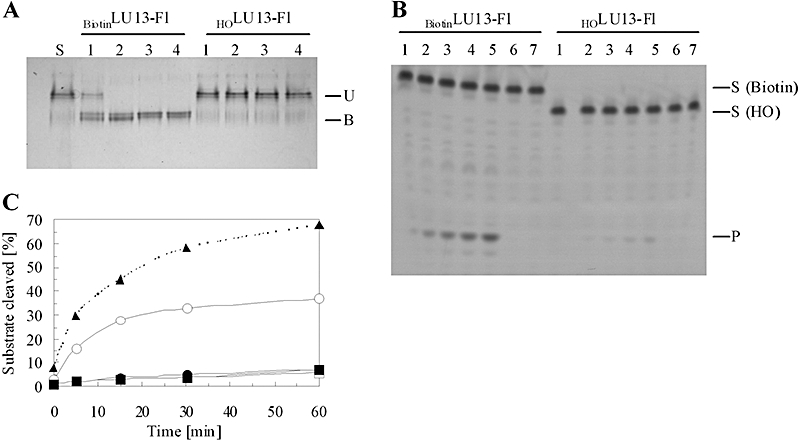
Assaying the cleavage of single-stranded segments linked via conjugation. A. The binding of 5′-biotinylated LU13 to streptavidin as monitored using native gel electrophoresis. The position of streptavidin was detected by staining with Coomassie blue. Labelling on the right indicates the position of unbound streptavidin (U) and streptavidin bound to 5′-biotinylated LU13 (B). Lanes 1–4 correspond to oligonucleotide amounts of 0.3, 0.6, 1.2 and 1.5 nmol respectively. The amount of streptavidin was 0.15 nmol in all the conjugation reactions. Lane S contains only streptavidin. 5′-hydroxylated LU13 was included as a control. B. The results of incubating 5′-biotinylated (Biotin) and hydroxylated (HO) LU13 with NTH-RNase E after mixing a fourfold molar excess with streptavidin (A). Lanes 1–5 correspond to samples removed after incubating with enzyme for 0, 5, 15, 30 and 60 min. The enzyme and substrate concentrations were 5 and 65 nM respectively. Lanes 6 and 7 correspond to samples incubated in reaction buffer without enzyme for 0 and 60 min respectively. The samples were separated on a denaturing 15% (w/v) polyacrylamide gel. C. Plots of the amount of product formed with time for the reactions shown in (B) and controls that were not mixed with streptavidin prior to incubating with NTH-RNase E (original gels not shown). Open and closed circles correspond to 5′-biotinylated and hydroxylated LU13, respectively, which had been preincubated with streptavidin, while the open and closed squares correspond to incubation of these substrates directly with NTH-RNase E. Closed triangles correspond to prewarmed 5′-monophosphorylated BR13. The substrate concentration was reduced to 65 nM (cf. Fig. 1) to slow the reaction, thereby permitting easier comparison with the 5′-monophosphorylated BR13 control.

The above experiments provided a clear indication that quadruplex formation was required for the phase of rapid cleavage; however, they did not distinguish between a requirement for the stacked G quartets *per se* or a substrate with multiple single-stranded regions. To investigate this further, we produced a substrate with multiple single-stranded regions by an alternative means. We biotinylated the 5′ end of LU13 (5′-GAGACAGU↓AUUUG), the BR13 G→A variant (see above) and then conjugated it to streptavidin (Fig. 4). Conjugation was monitored using native gel electrophoresis (Fig. 4A). No further shift in the mobility of streptavidin was observed when 5′-biotinylated LU13 was in fourfold excess (cf. lanes 3 and 4 with 2 on left of panel), which is consistent with each molecule of streptavidin having four binding sites for biotin (Chaiet and Wolf, 1964). We found, relative to a 5′-hydroxylated control, that 5′-biotinylated LU13 was cleaved more rapidly when conjugated to streptavidin prior to incubation with NTH-RNase E (Fig. 4B). In the absence of streptavidin conjugation, 5′-biotinylated LU13 was cleaved as poorly as its 5′ hydroxylated equivalent (Fig. 4C). From this study of well-characterized and relatively simple-oligonucleotide substrates, we concluded that the requirement for the rapid cleavage of a substrate that lacks a 5′ monophosphate is multiple single-stranded sites that can be recognized by RNase E. This predicts that reaction intermediates with a reduced number of intact sites would make poorer substrates. Consistent with this notion, a proportion of the 5′-biotinylated RNA that was conjugated with streptavidin appears to be resistant to rapid cleavage by RNase E (Fig. 4C).

### Quadruplexed RNA is bound with higher affinity

**Fig. 5:**
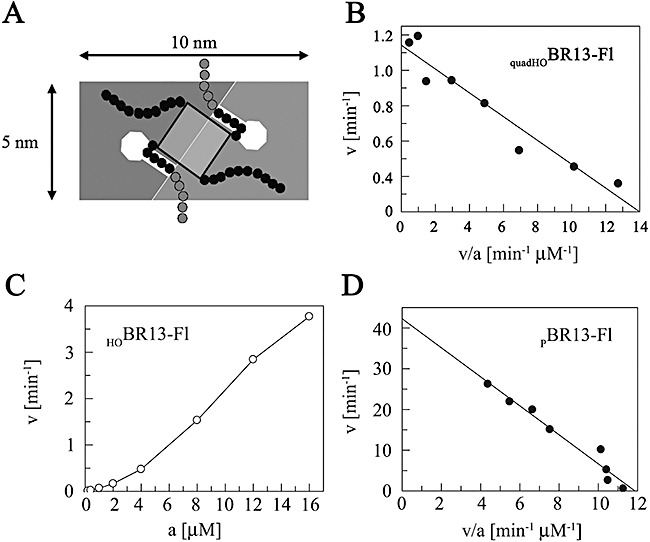
Quadruplexed RNA is bound with higher affinity. A. A schematic of quadruplex BR13 binding to a principal dimer of RNase E (top view). The two RNase E protomers that form the principal dimer are shown in dark and lighter grey. The 5′ sensor and the RNA-binding channel that leads to the catalytic site formed at the protomer–protomer interface are drawn as white octagons and rectangles respectively. The stacked G-quartets and nucleotides of the single-stranded tails of quadruplexed BR13 are represented by a transparent block and beads respectively. Two of the four single-stranded tails can bind the two RNA-binding channels of a principal dimer, and be cleaved at the active sites located at the interface between the protomers. The nucleotides that form the 3′ product of cleavage are coloured grey. The schematic is drawn to scale. Arrows indicate the width (10 nm) and depth (5 nm) of the principal dimer. B. The Michaelis–Menten analysis of quadruplexed, 5′-hydroxylated BR13 (_quadHO_BR13-Fl). Data obtained during cleavage assays were transformed into an Eadie-Hofstee plot. A linear fit to the data points is shown as a black line. This substrate was assayed over a concentration range of 25 nM to 4.0 μM using NTH-RNase E at a fixed concentration of 1 nM. The negative slope and the y-intercept of this plot represent the *K*_M_ and *k*_cat_ respectively. C. A Michaelis–Menten analysis for the cleavage of 5′-hydroxylated BR13 (_HO_BR13-Fl). Data are plotted as the initial rate (v) over substrate concentration (a). This substrate was assayed over a concentration range of 200 nM to 16 μM using 50 nM of NTH-RNase E. D. An Eadie-Hofstee plot for the cleavage of prewarmed 5′-monophosphorylated BR13 (_P_BR13-Fl). This substrate was assayed over a concentration range of 50 nM to 6 μM using 1 nM of NTH-RNase E.

Modelling studies using the known dimensions of structures of quadruplexes, streptavidin, single-stranded RNAs and the NTH of RNase E indicated that the two RNA-binding channels in a principal dimer of RNase E can simultaneously contact single-stranded regions in the context of either the G quadruplexes or streptavidin conjugates (Fig. 5). A schematic diagram of the former is provided (Fig. 5A). The duplication of contacts between macromolecules would be expected to increase greatly the affinity of the overall interaction. Consistent with this were the results of a Michaelis–Menten analysis. For the quadruplexed form of 5′-hydroxylated BR13, *K*_M_ and *k*_cat_ values were obtained of 0.13 μM and 1.9 min^−1^ respectively (Fig. 5B). These parameters were estimated from initial rates calculated only from time points during the period associated with the phase of rapid cleavage. For 5′-hydroxylated BR13 that had been prewarmed to dissociate the quadruplex, the *K*_M_ value is estimated to be > 8 μM. A *k*_cat_ value could not be determined because the reactions did not approach saturation at the highest substrate concentration, which was 16 μM (Fig. 5C). Thus, the formation of quadruplexes decreases the value of *K*_M_ by > 60-fold, which is consistent with a substantial increase in affinity. It is noteworthy that the curve obtained for prewarmed (and thus monomeric) 5′-hydroxylated BR13 appears sigmoidal, which is usually associated with positive cooperativity, a phenomenon that can be displayed by enzymes or receptors with multiple binding sites for a ligand. A sigmoidal curve and *K*_M_ of >8 μM were also obtained for 5′-hydroxylated LU13, which has the G to A substitution that prevents quadruplex formation (data not shown).

We also undertook a Michaelis–Menten analysis of 5′-monophosphorylated BR13 that had been prewarmed, the *K*_M_ and *k*_cat_ values that were obtained were 3.6 μM and 42 min^−1^ respectively (Fig. 5D). These values are in good agreement with those of a previous study (Redko *et al.*, 2003). Comparison of the values for 5′-monophosphorylated BR13 with those of the quadruplexed form of 5′-hydroxylated BR13 suggested that RNase E can bind with higher affinity to a 5′-hydroxylated substrate with multiple single-stranded regions than to a 5′-monophosphorylated substrate with one single-stranded site (*K*_M_ value of 0.13 versus 3.6 μM), but that this could be offset under non-saturating conditions by the reduction in the maximum rate of turnover (*k*_cat_ value of 1.9 versus 42 min^−1^). This is consistent with the quadruplexed form of 5′-hydroxylated BR13 being cleaved at a similar rate to that of 5′-monophosphorylated BR13 under our initial assay conditions (Fig. 1). It is possible that the maximum rate of turnover is reduced for RNase E cleavage of the quadruplexed form of 5′-hydroxylated BR13 because the predicted increase in the number of contacts with the RNA-binding channels results in product release being slower or because a 5′ monophosphate is required for the fastest rate of catalysis under saturating conditions or a combination of both.

### 5′-triphosphorylated transcripts can be cleaved rapidly by RNase E

**Fig. 6:**
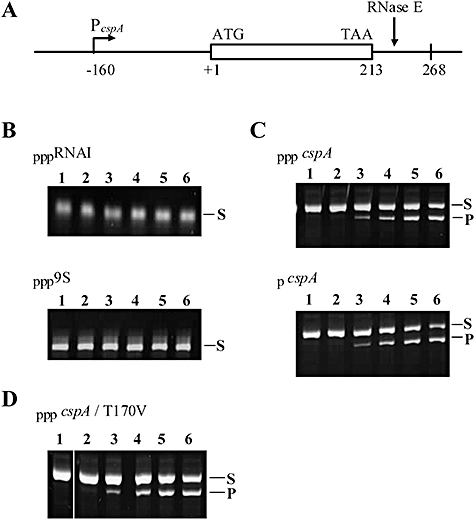
Cleavage of *cspA* mRNA by NTH-RNase E. A. A schematic diagram of the *cspA* gene. The vertical arrow indicates the major RNase E cleavage site in the 3′ untranslated region of the mRNA (position +234 as determined by primer extension). The bent arrow and vertical line indicate the position of the site of transcriptional initiation and termination respectively. Adapted from Hankins *et al.* (2007). B. The results of incubating 5′-triphosphorylated RNAI and 9S RNA with NTH-RNase E at substrate and enzyme concentrations of 180 and 5 nM respectively. The position of substrate, which was generated by *in vitro* transcription, is labelled S on the right of each panel. Where generated to a detectable level the major product is labelled P. Lane 1 corresponds to substrate incubated without enzyme for 60 min, while lanes 2–6 correspond to substrate incubated with NTH-RNase E for 0, 5, 15, 30 and 60 min respectively. C. As (B), except that the substrate is *cspA* mRNA with a triphosphate or monophosphate at the 5′ end. D. As (B), except that the substrate is 5′-triphosphorylated *cspA* incubated with the T170V mutant, which is defective in 5′ end sensing (Jourdan and McDowall, 2008). The samples were separated on denaturing 7% (w/v) polyacrylamide, sequencing-type gels and visualized by staining with ethidium bromide.

Having found that a model substrate can be cleaved rapidly by the NTH of RNase E independent of interaction with a 5′ monophosphate, we extended our analysis to transcripts of *E. coli* (Fig. 6). Included was *cspA* mRNA, whose cleavage by a degradosome preparation had been found not to be enhanced substantially by the conversion of the 5′ group from a tri- to monophosphate (Hankins *et al.*, 2007). The major site of cleavage is on the 5′ side of the transcriptional terminator at the 3′ end (Fig. 6A). In comparison with 5′-triphosphorylated RNAI and 9S RNA, which formed the basis of previous studies of RNase E (Bouvet and Belasco, 1992; McDowall *et al.*, 1994; Mackie, 1998; Kaberdin *et al.*, 2000; Walsh *et al.*, 2001) and were included here as controls (Fig. 6B), we found that *cspA* mRNA can be cleaved rapidly irrespective of the phosphorylation status of its 5′ end (Fig. 6C). Indeed, sufficient turnovers (∼20) occurred during a typical assay that the major upstream product was detectable by staining with ethidium bromide. Thus, in contrast to a recent commentary (Schoenberg, 2007), the presence of a 5′ monophosphate is not required for the rapid cleavage of all *E. coli* RNAs by RNase E. Consistent with this, the mRNA of *cspA* was still cleaved rapidly when incubated with the 5′ end-sensing T170V mutant of NTH-RNase E (Fig. 6D). Relative to wild-type, T170V cleaves 5′-monophosphorylated BR13 > 15-fold slower, without an obvious effect on the rate of cleavage of the 5′-hydroxylated equivalent (Fig. S1). As found above for the quadruplexed form of BR13 (Fig. 1), rapid cleavage of *cspA* mRNA lacking a 5′ monophosphate is a property of the NTH of RNase E (Fig. 6). The ability to cleave 5′-triphosphorylated *cspA* at a rate similar to its monophosphorylated equivalent appears not to be diminished within the context of the degradosome (Hankins *et al.*, 2007).

**Fig. 7:**
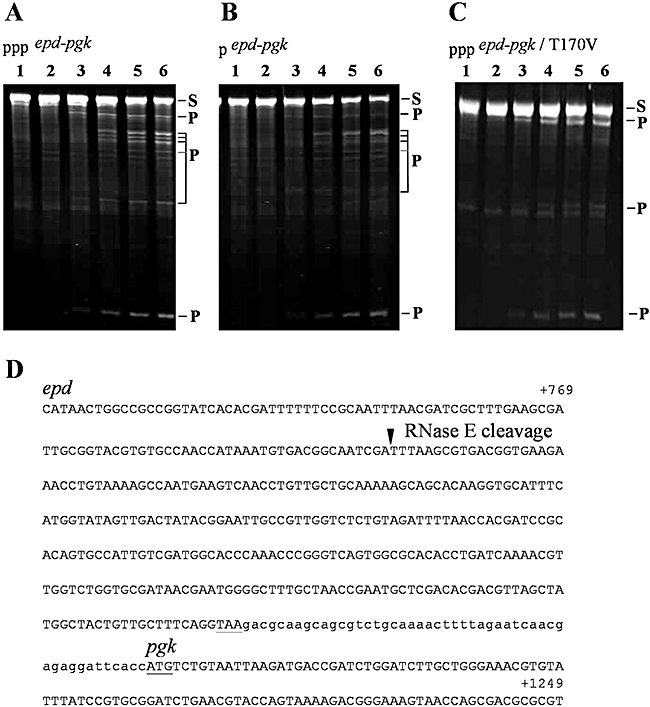
Cleavage of *epd-pgk* by NTH-RNase E. A and B. The results of incubating NTH-RNase E with 5′-triphosphorylated and 5′-monophosphorylated *epd-pgk* RNA respectively. C. The results of incubating 5′-triphosphorylated *epd-pgk* RNA with the T170V mutant of NTH-RNase E. The reaction conditions and labelling are as Fig. 6. The above samples were separated on denaturing 6% (w/v) polyacrylamide gels and visualized by staining with ethidium bromide. D. The position of the major site in 5′-triphosphorylated *epd-pgk* RNA cleaved by the T170V mutant. The stop and start codons of *epd* and *pgk*, respectively, are underlined and the intergenic region is in lower case. The positions are numbered according to the transcription initiation point of the P_0_ promoter upstream of *epd*. This site was mapped by cloning and sequencing the 3′ fragment by RNA ligase-mediated reverse transcription PCR (Kime *et al.*, 2008) and the 5′ fragment using standard reverse transcription and PCR (see *Experimental procedures* for details).

We also analysed the cleavage of *epd-pgk* RNA by RNase E (Fig. 7) as it had been suggested that this transcript might be an example of internal entry (Bardey *et al.*, 2005), but to our knowledge the rate of cleavage of this substrate had not been compared directly with others and its 5′ monophosphate independence had not been proven experimentally. We found that degradation of the *epd-pgk* transcript (Fig. 7A) is not stimulated greatly when its 5′ end is monophosphorylated (Fig. 7B). Thus, the initial cleavage of this substrate appears to be largely independent of interaction with a 5′ monophosphate. However, not all of the products resulted from 5′ monophosphate-independent cleavage: incubation of the T170V mutant of RNase E with 5′-triphosphorylated *epd-pgk* RNA produced a reduced number of fragments (Fig. 7C). Analysis of these products using RNA ligase-mediated reverse transcription PCR (Kime *et al.*, 2008) revealed a 5′ and 3′ end that correspond to a single position within the 3′ end of the coding region of *epd* (Fig. 7D). This likely represents the major site of 5′ monophosphate-independent cleavage. Cleavage at this site is predicted to produce the smallest and largest fragments, which are also the most abundant. The cleavage that produces a detectable fragment of intermediate size has yet to be identified. The overall rate of RNase E cleavage of 5′-triphosphorylated *epd-pgk* was faster than 5′-triphosphorylated 9S RNA and RNAI, but not as fast as the rate of cleavage of 5′-triphosphorylated *cspA* mRNA (cf. Fig. 6).

### RNase E recognizes multiple single-stranded regions in *cspA* mRNA

**Fig. 8:**
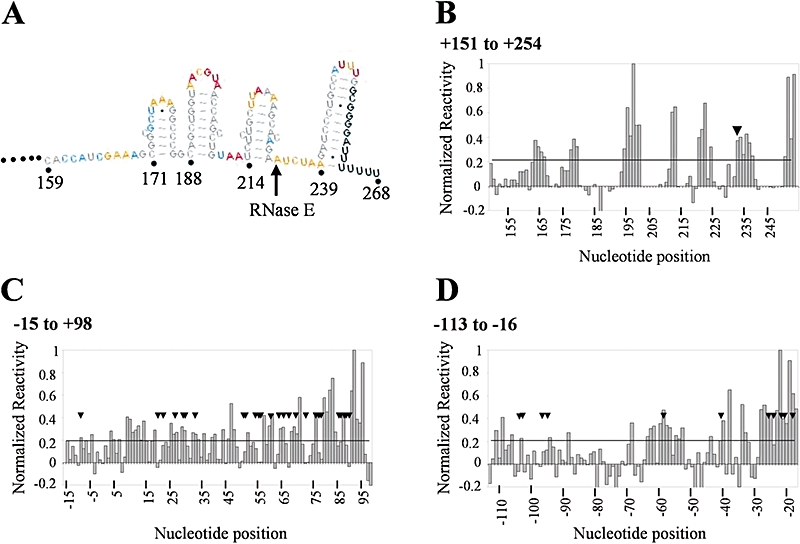
Mapping of single-stranded regions in *cspA* mRNA recognized by NTH-RNase E. Single-stranded regions of *cspA* mRNA were mapped using NMIA modification data as constraints. A. A representative structure prediction for the region between nucleotides +159 and +268 that contains the major site of RNase E cleavage. The degree of NMIA modification of individual nucleotides is represented on a colour scale where red, orange, blue and grey represents high (50–100%), medium (25–49%), low (12–24%) and insignificant (0–11%) reactivity respectively. Nucleotides with reactivities between 25% and 100% were constrained to be single-stranded. The nucleotides for which no data were collected are represented by black letters. B, C and D. The normalized reactivity of each nucleotide position in three regions of *cspA* mRNA. Sites of cleavage that were detected after incubation with the D346N mutant of NTH-RNase E are indicated by closed triangles.

Next to investigate whether RNase E recognizes single-stranded segments in addition to a major site of cleavage, *cspA* mRNA was probed using the selective acylation of 2′-hydroxyl groups (Merino *et al.*, 2005; Wilkinson *et al.*, 2006) in the absence and presence of D346N, an active site mutant of RNase E (Callaghan *et al.*, 2005a). The sensitivity of nucleotides to acylation, which is associated with single-strandedness, was quantified using semi-automated footprinting analysis software (Das *et al.*, 2005; Laederach *et al.*, 2008). The relative sensitivity at every position within three probed regions of *cspA* mRNA is shown using histograms (Fig. 8). The raw images are provided as supplementary data (Fig. S2). Comparison of the sensitivity data with known structures within the 3′ end of *cspA* mRNA (Fig. 8A) revealed that a value of ≥ 0.2 is indicative of a single-stranded region. Numerous segments with a value of ≥ 0.2 were identified in each of the three regions covered by our primer extension analysis (Fig. 8B–D).

We initially hoped that the activity of the D346N mutant would be sufficiently low to detect ‘footprints’ corresponding to sites of binding. However, at the micromolar concentrations of enzyme required to ensure that the majority of the RNA was bound, cleavage occurred to a detectable level: at several positions the primer extension reactions terminated independent of acylation (Fig. S2). In addition to the major cleavage site in the 3′ UTR of *cspA*, we mapped multiple secondary sites in the 5′ UTR and protein-coding region. The positions at which cleavage occurred are indicated by inverted triangles in the histograms (Fig. 8). Consistent with the primer extension analysis, numerous products in addition to those corresponding to cleavage at the major site in the 3′ UTR were detected when the products of incubating *cspA* mRNA with micromolar instead of nanomolar concentration of NTH-RNase E were analysed by gel electrophoresis (data not shown). Although footprints were not obtained using the acylation assay, the mapping of sites of secondary cleavage confirmed that NTH-RNase E can recognize multiple single-stranded segments within *cspA* mRNA. The contribution of the individual single-stranded sites to the rapid cleavage of *cspA* mRNA in its 3′ UTR is currently being investigated.

## Discussion

Using derivatives of BR13 (Figs 1–5) and transcripts (Figs 6 and 7), we have shown for the first time that *E. coli* RNase E can cleave rapidly RNAs that lack a 5′ monophosphate group. Rapid cleavage of BR13 was facilitated by forming substrate multimers that modelling studies showed would allow simultaneous contacts between two single-stranded segments and two RNA-binding channels in the principal dimer of tetrameric RNase E (Fig. 5). The multimers were formed by joining 5′-hydroxylated BR13 via G quadruplex formation (Fig. 3) or a 5′-biotinylated derivative via streptavidin conjugation (Fig. 4). Michaelis–Menten analysis revealed that the value of *K*_M_ for RNase E cleavage of the quadruplexed form of 5′-hydroxylated BR13 was >60 fold lower than that of its monomeric equivalent (Fig. 5). Based on the above, our working model is that the simultaneous interaction of two or perhaps more single-stranded segments with RNase E can negate the requirement for a 5′ monophosphate group.

The mRNA transcripts shown here to be cleaved rapidly by RNase E in the absence of interaction with a 5′ monophosphate were monocistronic *cspA* mRNA (Fig. 6) and dicistronic *epd-pgk* mRNA (Fig. 7). RNase E cleaves *cspA* mRNA at a site located just upstream of the transcriptional terminator. In accordance with our biochemical analysis (Fig. 6), *cspA* mRNA in *E. coli* does not appear to accumulate in the absence of the RppH 5′ pyrophosphatase (Deana *et al.*, 2008), but does when RNase E is inactivated (Fang *et al.*, 1997; Hankins *et al.*, 2007). RNase E cleavage of *cspA* mRNA is thought to accelerate degradation by removing a 3′ stem-loop that belongs to a class of structures known to impede the progress of 3′ exonucleases (for review, see Belasco and Higgins, 1988; Regnier and Arraiano, 2000; Andrade *et al.*, 2009a). The paradigm for this mode of mRNA degradation is *rpsO* mRNA (Regnier and Hajnsdorf, 1991; Braun *et al.*, 1998; Andrade *et al.*, 2009b). The cleavages we mapped for the *epd-pgk* transcript *in vitro* (Fig. 7) may also mirror the situation in *E. coli*. Others have identified decay intermediates in *E. coli* that correspond to cleavage within the *epd* coding region and have provided evidence that the translation of *epd* is poor relative to other *E. coli* transcripts (Bardey *et al.*, 2005). Thus, it appears that the amount of ribosome traffic on *epd* mRNA is insufficient to completely block access by RNase E (for review, see Dreyfus, 2009).

We propose that single-stranded sites in *cspA* and *epd* mRNA, other than those that are cleaved, contribute to the binding of RNase E. This was investigated for *cspA* mRNA. Incubation of this transcript with concentrations of RNase E vastly exceeding that required for cleavage in the 3′ UTR identified secondary sites within several single-stranded regions (Fig. 8). It should be noted that sites that are cleaved poorly may still be bound with high affinity. Evidence of such has been obtained recently by us for RNase G (S.S. Jourdan and K.J. McDowall, unpubl. result). As multiple single-stranded segments are ubiquitous within the coding regions of mRNA transcripts, our model offers a simple explanation for the finding that in the absence of translating ribosomes many transcripts are highly susceptible to RNase E (Dreyfus, 2009). We suggest that this may also hold when translation is blocked by the binding of small antisense RNAs (for reviews, see Gottesman, 2005; Repoila and Darfeuille, 2009; Waters and Storz, 2009). While it has been reported that small antisense RNAs can physically recruit RNase E as part of the degradosome (Morita *et al.*, 2005), this might not be required generally for the rapid degradation of targeted mRNA.

An issue arising from the finding that *cspA* and *rpsO* mRNA appear to be degraded 3′ exonucleolytically from an RNase E site downstream of the coding sequence is the opportunity for premature translational termination, which would be wasteful in terms of ribosome usage and the energy used to make truncated polypeptides most of which will have no function. However, should it transpire that efficient RNase E cutting of *cspA* and *rpsO* mRNA requires the recognition of a site within the coding region, this would be expected to minimize the degradation of those transcripts that are being highly translated. Stochastic variation in translation initiation may ensure that all mRNA are eventually susceptible to RNase E, while individual transcripts that are defective in translation would be expected to be degraded rapidly.

We envisage that single-stranded segments recognized simultaneously by RNase E could be at distance in terms of sequence length and separated by structured regions, could work cooperatively with a 5′ monophosphate when accessible and could involve contacts with both of the principal dimers of tetrameric NTH-RNase E. Moreover, one or more of the single-stranded sequences recognized by RNase E could be presented as part of a secondary structure, e.g. as a bulge or loop within a stem-loop. The first of the above possibilities might explain why the deletion of the first 71 nt of *cspA*, which contains several secondary sites recognized by RNase E (Fig. 8), reduces the rate of RNase E cleavage of *cspA* mRNA *in vitro* (Fig. S3), while the last possibility might be of relevance to the recent finding that RNase E autoregulates its production in *E. coli* by a mechanism that involves the binding of a stem-loop within the 5′ UTR of its gene (Schuck *et al.*, 2009). It is also possible that some substrates may have 3D structures in which single-stranded segments are pre-aligned to interact simultaneously with RNase E: this would be expected to lower the entropic barrier to complex formation and enhance the rate of cleavage. It is well documented that the conformational context of sites cleaved by RNase E has a role in determining the efficiency of cleavage (for review, see Coburn and Mackie, 1999).

In summary, we have shown that for at least some substrates rapid cleavage by RNase E can occur regardless of the phosphorylation status at their 5′ end. This provides an explanation for the finding that bulk mRNA degradation is affected less by disruption of RppH than RNase E (Deana *et al.*, 2008). Moreover, as multiple single-stranded segments in a conformational context that permits their simultaneous interaction with RNase E may occur frequently in mRNA transcripts, our work suggests that direct entry by RNase E (Baker and Mackie, 2003) could represent a major pathway for the initiation of mRNA degradation in *E. coli* and other organisms that contain homologues of this enzyme. An important question that remains to be addressed is why some initial RNase E cleavages in *E. coli* are dependent on the generation of a 5′-monophosphorylated end, while others are not. It is possible that a 5′-monophosphorylated end (in the absence of terminal base-pairing) provides an important foothold for RNase E when there is a paucity of single-stranded segments that can facilitate the binding of RNase E. Such is most likely to be the case for ribosomal RNA precursors that are highly structured and associate with ribosomal proteins as well as for mRNAs that are translated efficiently and have short or highly base-paired UTRs. For some transcripts, it might be that RNase E can bind in the absence of a 5′ monophosphate, but that the presence of this group is required to position RNase E on the RNA at a site that can be cleaved. The latter might explain why 5′-triphosphorylated and circularized *rpsT* mRNA appears to be cleaved poorly by RNase E *in vitro* despite having multiple single-stranded regions (Mackie, 1998).

## Experimental procedures

### Purification of NTH-RNase E and assay conditions for RNA cleavage

Recombinant, oligohistidine-tagged polypeptides corresponding to the NTH of RNase E with wild-type or mutant sequences were purified as described previously (Callaghan *et al.*, 2003; Callaghan *et al.*, 2005b) and stored at −80°C in 20 mM Tris-HCl (pH 7.6), 500 mM NaCl, 10 mM MgCl_2_, 10 mM DTT, 0.5 mM EDTA, and 5% (v/v) glycerol (Jourdan and McDowall, 2008). Protein concentrations were established using a modified Bradford assay (Bio-Rad) and SDS-polyacrylamide gel electrophoresis. The cleavage assays were done as described previously (Redko *et al.*, 2003; Jourdan and McDowall, 2008). The oligonucleotide substrates labelled with fluorescein at the 3′ end were synthesized and purified by Dharmacon (USA). The sequences of BR13 and LU13 were 5′-GGGACAGU↓AUUUG and 5′-GAGACAGU↓AUUUG respectively. Initial rates were obtained by establishing the slope representing percentage product generated over time during the initial, linear phase of the reaction. The rate calculations took into account the concentration of enzyme and substrate in the reaction mix.

### Synthesis of RNA transcripts

Transcripts were synthesized *in vitro* using T7 RNA polymerase and PCR-generated templates and purified as described previously (Kaberdin *et al.*, 2000; Walsh *et al.*, 2001). When required monophosphate groups were added at the 5′ end by treating with Calf Intestinal Phosphatase (New England Biolabs) then T4 polynucleotide kinase (New England Biolabs) as described previously (Kime *et al.*, 2008). The sequences of the primers used to generate the templates were 5′-CGCAGAATTCTAATACGACTCACTATAGGGTTTGACGTACAGACC plus 5′-AAAATCCCCGCCAAATGGCAGGG for *cspA*, 5′-GGATCCTAATACGACTCACTATAGGGACAGTATTTG plus 5′-AACAAAAAAACCACCGCTACC for RNAI, 5′-CGCAAGCTTTAATACGACTCACTATAGGGAAGCTGTTTTGGCGGATGAG plus 5′-GTCGCGTCGACACGAAAGGCCCAGTCTTTC for 9S RNA, and 5′-ATCCTAATACGACTCACTATAGGGTTATTGATGCATACC plus 5′-TCAACGCCGTCGAGGTAATC for *epd-pgk*. The T7 polymerase promoter in each of the forward primers is underlined.

### Conjugation of 5′-biotinylated oligonucleotides to streptavidin

Increasing amounts of 5′-biotinylated LU13 (0.15, 0.3, 0.6 and 1.5 nmol) were incubated with streptavidin from *Streptomyces avidinii* (Sigma) (0.15 nmol) in 100 μl of RNase E reaction buffer (Redko *et al.*, 2003; Jourdan and McDowall, 2008) containing 80 U of RNaseOUT (Invitrogen) at 30°C for 20 min. Samples of the reaction products were added to an equal volume of loading buffer [100 mM Tris-HCl, pH 6.8, 20% (v/v) glycerol and 0.2% (w/v) bromophenol blue] and analysed by native gel electrophoresis using 12% (w/v) 29:1 acrylamide : *bis*-acrylamide gels containing 150 mM Tris-HCl, pH 6.8 in the upper stacking gel and 375 mM Tris-HCl, pH 8.8 in the resolving gel and electrophoresis buffer containing 192 mM glycine and 25 mM Tris-HCl, pH 8.3. Gels were stained using 0.2% (w/v) Coomassie Brilliant Blue R in 50% (v/v) methanol and 7% (v/v) glacial acetic acid and proteins visualized following destaining with 20% (v/v) methanol and 7% (v/v) glacial acetic acid. Products from the reaction with the lowest concentration of 5′-biotinylated RNA that shifted all of the streptavidin were used in subsequent experiments. This corresponded to an RNA concentration of 0.6 nmol, which was fourfold higher than the streptavidin concentration.

### CD spectroscopy

RNAs were analysed by circular dichroism at a concentration of 7.5 μM in 25 mM *bis*-Tris-propane (pH 8.3), 100 mM NaCl. Measurements were made using a Jasco J-715 spectropolarimeter equipped with a Peltier temperature controller (Jasco PTC 351S). Samples of 240 µl were placed in a quartz cuvette with an optical path length of 1 mm. After transfer to the spectropolarimeter, the sample was allowed to equilibrate at 4°C for 10 min. Five CD scans were performed at 50 nm min^−1^ with a 2 s response time, 1 nm pitch and 1 nm bandwidth over the wavelength range of 220–320 nm. The sensitivity was set to 10 mHg and the slit width to 1000 μm. The average of the five scans was taken. Then the temperature was raised to 37°C and the sample was allowed to equilibrate for 15 min before scanning at this temperature. For each temperature, a CD spectrum of the buffer was recorded and subtracted from the spectrum obtained for the sample containing RNA. The high tension values were below 400 during all CD experiments, indicating that the obtained data have a high signal to noise ratio and are reliable. Data were zero-corrected at 320 nm. After all the measurements were made, the contents of the cuvette were analysed by denaturing gel electrophoresis to confirm the concentration and integrity of the RNA.

### Modelling studies

Potential molecular interactions were visualized *in silico* using RasMol (http://www.umass.edu/microbio/rasmol/). The PDB files for the NTH of RNase E and quadruplexes were 2c0b (plus 2c4r) and 1S9L (plus 2GRB) respectively.

### SHAPE analysis

Probing using selective acylation of 2′-hydroxyl groups was carried out essentially as described previously by others (Wilkinson *et al.*, 2006). RNA was transcribed such that it had additional sequences at its 5′ and 3′ ends to allow the extremes of the natural transcript sequence to be probed. The sequences of the primers used to generate the template for extended *cspA* mRNA were 5′-ATCCTAATACGACTCACTATAGGGCCTTCGGGCCAAGGTTTGACGTACAGACC and 5′-GAACCGGACCGAAGCCCGATTTGGATCCGGCGAACCGGATCGAAAAATCCCCGCCAAATGG.

RNA (1 pmol) was incubated in 25 mM *bis*-Tris-Propane (pH 8.3), 100 mM NaCl, 15 mM MgCl_2_, 0.1% (v/v) Triton X-100, 2.5% (v/v) glycerol and 1 mM DTT in the presence or absence of up to 12 μM of D346N NTH-RNase E. After incubating for 20 min, NMIA (N-methylisatoic anhydride) dissolved in DMSO was added to a final concentration of 13 mM and incubation continued at 37°C for a further 45 min. A negative control was included in which DMSO without NMIA was added. The RNA was then recovered by ethanol precipitation as described previously (Kime *et al.*, 2008).

Primer, radiolabelled at the 5′ end using T4 polynucleotide kinase, was added to the modified RNA that had been heated to 95°C for 3 min. Annealing was achieved by incubating at 65°C for 5 min, 35°C for 5 min and then placing on ice for 1 min. Sequencing was performed using unmodified RNA and dideoxyribonucleotide triphosphates that were added to a final concentration of 0.75 mM. The primer was extended in a buffer containing 75 mM KCl, 50 mM Tris-HCl (pH 8.3), 3 mM MgCl_2_, 0.5 mM of each deoxyribonucleotide triphosphate and 5 mM DTT. The reaction was warmed to 55°C before addition of 200 U of Superscript III reverse transcriptase (Invitrogen) and incubated for a further 30 min at 55°C. The final volume of the reaction was 20 μl. After reverse transcription, the RNA was degraded by adding NaOH to a final concentration of 200 mM and incubating at 95°C for 5 min. Next to be added was 29 μl of a 4:25 (v/v) mixture of 1 M unbuffered Tris-HCl: stop dye [85% (v/v) formamide, 0.5× TBE, 50 mM EDTA, pH 8.0, 0.025% bromophenol blue and 0.025% xylene cyanole].

The samples were incubated at 95°C for 5 min before being analysed in denaturing polyacrylamide gels: 7 M urea, 8–10% (w/v) 29:1 acrylamide : *bis*-acrylamide, 1× TBE. Extended products were detected using a Phosphorimager FLA-5100 (FUJIFILM) with the laser set to 635 nm. Band intensity was quantified using the semi-automated footprinting analysis programme (Das *et al.*, 2005; Laederach *et al.*, 2008). Absolute NMIA reactivity at each band position was calculated by subtracting the (−) NMIA intensities from the (+) NMIA intensities. For each primer that was extended, the average intensity of the bands in the ddATP sequencing lane was used to correct for the number of counts loaded and values were then normalized to the highest peak set with a value of 1. Secondary structure predictions of *cspA* were obtained using the RNA structure programme (Mathews *et al.*, 2004) using the chemical modification constraint. RNA secondary structure diagrams were drawn using xrna (http://rna.ucsc.edu/rnacenter/xrna/). The sequences of the primers used in the analysis of *cspA* structure were 5′-GAACCGGACCGAAGCCCG, 5′-CCATCGTTCTGGATAGCAGAG and 5′-CATTTTACCGGACATAGTGTATTACC.

### Identification of 5′-monophosphorylated and 3′-hydroxylated ends

RNA samples were treated with poly(A) polymerase before ligation with an RNA adapter, which only ligates to a 5′-monophosphorylated end, as described previously (Kime *et al.*, 2008). Following reverse transcription using an oligo (dT) primer (5′-TTTTTTTTTTTTTTTTTTTTTTTTTTTTT(G/C/A) or random hexamers (Amersham Biosciences), the products were amplified by PCR and cloned into the pGEM-T Easy vector for sequencing of the amplicons. A number of primer combinations were used. An oligonucleotide (5′-CATGAGGATTACCCATGTCG) complementary to the RNA adapter was used in combination with reverse primers that bound at different positions in the transcript to amplify fragments corresponding to 5′-monophosphorylated ends. The oligo (dT) primer in combination with a forward primer bound at the 5′ end of the transcript to amplify fragments corresponding to 3′ hydroxylated ends. The sequence of a reverse primer that produced an amplicon that identified the 5′-monophosphorylated end generated by RNase E cleavage of *edp-pgk* RNA was 5′-AAGTTTTGCAGACGCTGCTT. The sequence of the forward primer that produced an amplicon that identified the 3′ hydroxylated end generated by RNase E cleavage was 5′-ATCCTAATACGACTCACTATAGGGTTATTGATGCATACC.

## Note added in proof

Since submitting our manuscript, others have found that the ability of RNase E to interact strongly with a 5′ monophosphate group is not required for *E. coli* viability (Garrey *et al.*, 2009). Like our work report here, this points to the importance of 5′ monophosphate-independent RNA recognition.
