# Supplementary material for: Rapid cleavage of RNA by RNase E in the absence of 5′ monophosphate stimulation
Source: Mol Microbiol. 2009 Nov 18;76(3):590–604. doi: 10.1111/j.1365-2958.2009.06935.x (PMC2948425; doi:10.1111/j.1365-2958.2009.06935.x)
Supplement: Supplementary file 1 [file mmi0076-0590-SD1.pdf]

Supplementary material

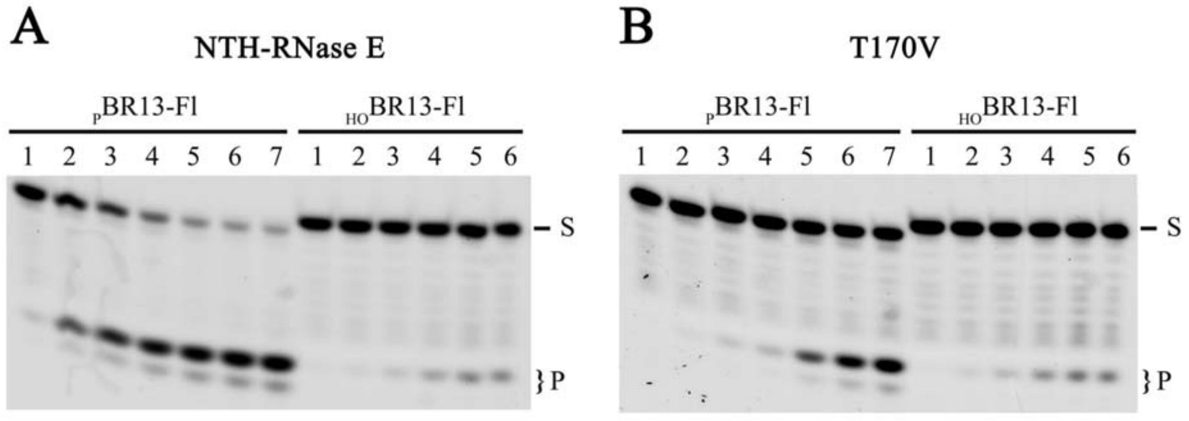

Figure S1. Properties of the T170V mutant. The assay of wild-type NTH-RNase E and the 5’-sensor mutant T170V are shown in panel **A** and **B**, respectively. Both 5’-monophosphorylated and 5’-hydroxylated BR13 were used as substrates. The concentrations of enzyme and substrate in each reaction were 5 nM and 250 nM, respectively. For $_{P}$BR13-Fl, lanes 1 to 7 correspond to time points of 0, 2, 5, 10, 50, 120, and 180 min, respectively. For $_{HO}$BR13-Fl, lanes 1 to 6 correspond to samples taken at 0, 10, 50, 120, 180 and 330 min, respectively. All of the substrates were prewarmed to dissociate quadruplexes prior to starting the reactions. The substrate and product band(s) are indicated to the right of the panels.

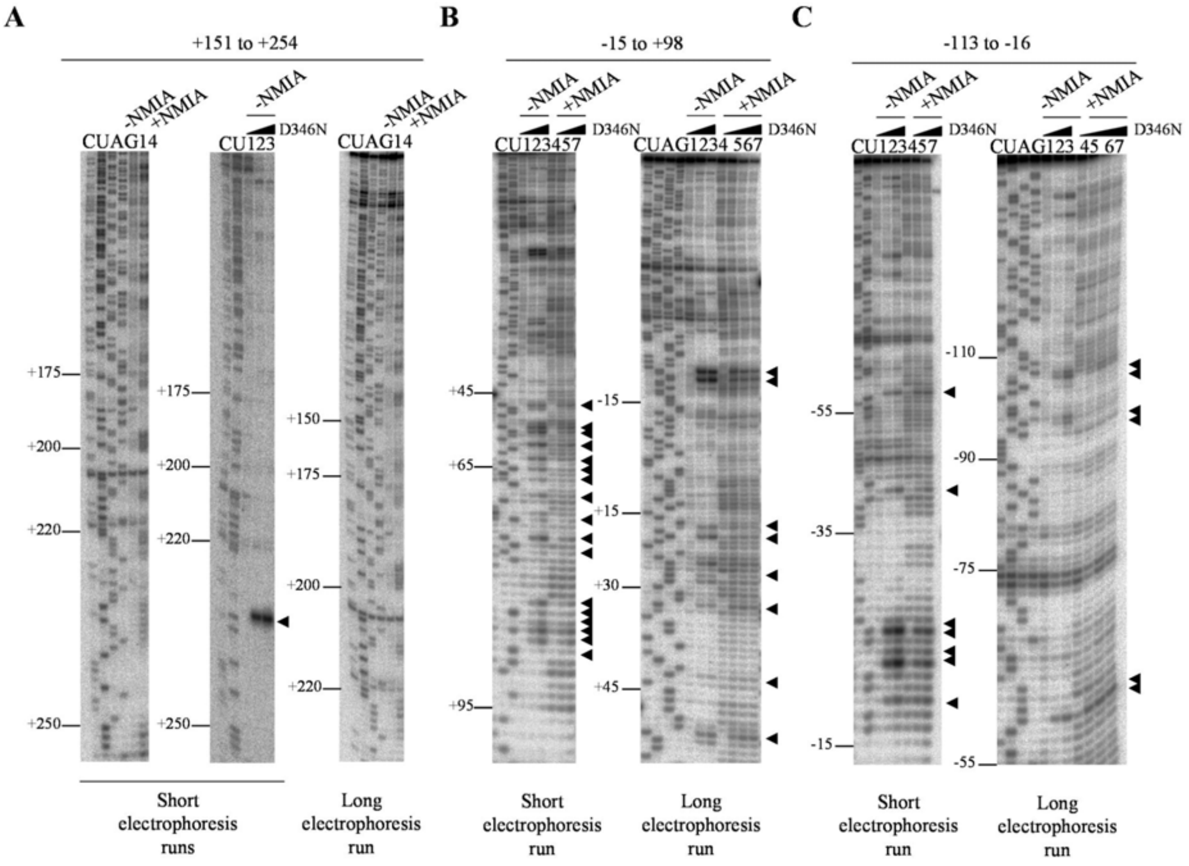

Figure S2. **SHAPE analysis of the *cspA* mRNA**. Sequencing gels are shown for the primer extension analysis of regions of *cspA* between +151 to +254 (panel **A**), -15 to +98 (panel **B**) and -113 to -16 (panel **C**). Lanes represent unmodified RNA (lanes 1-3) and NMIA modified RNA (lanes 4-7) in the absence (lanes 1 and 4) and presence of 3 µM (lanes 2 and 5), 6 µM (lane 6) or 12 µM (lanes 3 and 7) of the D346N mutant of NTH-RNase E. Dideoxy-sequencing reactions are represented by lanes labelled C, U, A and G. Short and long electrophoresis runs are indicated beneath the panels. Numbering to the left of each panel indicates the nucleotide number within the *cspA* mRNA sequence and RNase E cleavage sites are indicated on the right of each panel by closed triangles.

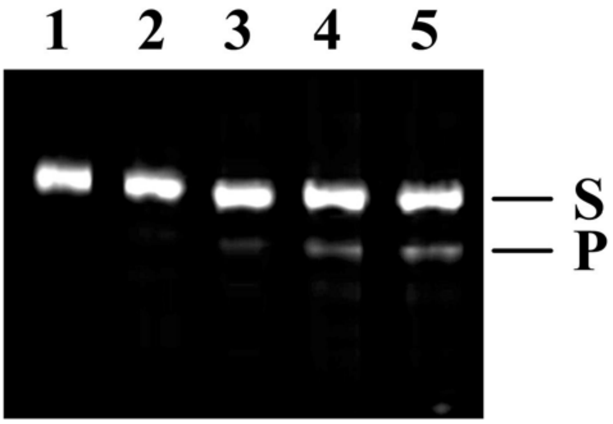

Figure S3. Cleavage of *cspA* mRNA lacking the first 71 nucleotides. The first nucleotide of this truncated substrate is -89 relative to the translation start codon. The enzyme and substrate concentrations were 5 and 180 nM, respectively. The position of the substrate (S) and upstream product (P) is indicated on the right of the panel. Lanes 1 to 5 correspond to substrate incubated with wild-type NTH-RNase E for 0, 5, 15, 30 and 60 min, respectively.
